# Slope stability calculation method for highwall mining with open-cut mines

**DOI:** 10.1038/s41598-021-04130-w

**Published:** 2022-01-07

**Authors:** Juyu Jiang, Ye Lu, Dong Wang, Xinping Han

**Affiliations:** College of Mines, Liaoning Technical University, Fuxin, 123000 Liaoning China

**Keywords:** Environmental sciences, Natural hazards, Solid Earth sciences

## Abstract

Slope stability is a prominent problem for the efficient application and promotion of highwall mining technology, especially when mining residual coal under high and steep end-slope conditions. This study proposes the concept of target time pillar strength based on the required coal pillar service time. Creep tests were performed to measure the time-varying properties of coal shear strength parameters under different loads, and a time-varying function was established by regression. The highwall mining length is divided into three categories based on discontinuous structural plane theory, including goaf, yielding, and elastic zones, all of which are considered to have resistances against shear stress. The basal coal seam is prone to weakening owing to the weight of overlying strata, which may shift the slope failure mode from circular to sliding along the weak layer. Numerical modeling was used to study the influence of the bearing stress and target time strength on the development of the yielding zone at the coal pillar ribs. The coefficients of the three zones were determined, and the temporal and spatial evolution patterns of the shear strength parameters of the weak layer were acquired. A slope stability calculation method is proposed based on rigid body-limit equilibrium theory that can quantify the influence of highwall mining operations on slope stability, which is significant for popularizing highwall mining technology.

## Introduction

China has approximately 170 billion tons of open-cut mining reserves and roughly 30 open-cut coal mines operating at production scales of more than 10 Mt/a, some of which exceed 30 Mt/a^1^. However, open-cut mining inevitably leaves a significant amount of retained coal at the base of the highwall and endwall above the floor. Preliminary estimates have suggested that there are as much as 20 billion tons of retained coal, which lowers the recovery rate and not only leads to economic loss in coal mines but also geotechnical and environmental hazards (e.g., spontaneous combustion, slope instability). Coal extraction from basal coal seam ribs has gained popularity in recent years with the development and improvement of highwall mining machines, and highwall mining has gradually become the norm in open-cut coal mines in China^2^.

Highwall mining combines the advantages of open-cut and underground approaches, and allows soft and thick coal seams to be mined at the rear of the coal rib at the highwall/endwall toe. The entire mining process is automated and controlled remotely to achieve unmanned intelligent extraction and transportation. Coal pillars should be left between each highwall channel to support the overlying strata to prevent slope instability and machine burial. A slope stability calculation method is therefore required to determine the appropriate coal pillar parameters. A significant body of research^3–7^ has attempted to develop reliable methods to determine coal pillar parameters using empirical formulas, laboratory tests, analytical solutions, and numerical simulations. For example, Chen et al.^8^ investigated the influence of coal pillar parameters on slope stability based on the bearing capacity model and catastrophe theory, and proposed a slope stability failure criterion that includes the coal pillar parameters. Wang et al.^9^ studied coal pillar capacity under highwall mining operations using catastrophe theory, time-dependent (creep) laboratory tests, and numerical simulations, and determined the appropriate coal pillar width for highwall mining. Wang et al.^10^ revealed the failure mechanism and process of coal pillars under highwall mining based on the stress distribution at the pillar rib, as derived from the I-II mixed mode crack model from fracture mechanics and failure criteria. These studies have enriched the knowledge base of coal pillar failure mechanisms and parameters under highwall mining operations and resolved complicated coal pillar design issues.

A weak layer readily forms on the edge of the highwall mining area owing to the weight of overlying strata, which reduces the slope stability. The slope and coal pillar stabilities are interrelated, making it important to simultaneously prevent their failure. Numerous slope stability studies have involved highwall mining^11–16^. For example, Ross et al.^17^ developed the Slope-W model based on steep slope analysis and implemented a practical method. The Slope-W model proposes a highwall mining sequence for thick coal seams that starts from top to bottom of the seam and improves the overall slope stability. Chandar et al.^18^ performed numerical simulations to study the influence of mining length, pillar width, and highwall channel number on slope stability, and categorized slope stability scenarios using multi-linear regression, logistic regression, and naive Bayes classification methods. However, general agreement has not been met regarding the influence of coal pillar parameters on slope stability^19,20^ primarily owing to a lack of proven slope stability calculation methods for highwall mining. A series of time-dependent laboratory studies on coal pillars is therefore undertaken herein to investigate the potential failure mechanism of slopes under highwall mining operations based on discontinuous structural plane theory^21^. The extent of plastic deformation at the rib of the coal pillars is investigated under various mining length, supporting capacity, and time-dependent strength conditions. A slope stability calculation method for highwall mining with time-dependent behavior is proposed, which provides meaningful insight for Chinese highwall mining technology.

## Engineering background

**Figure 1:**
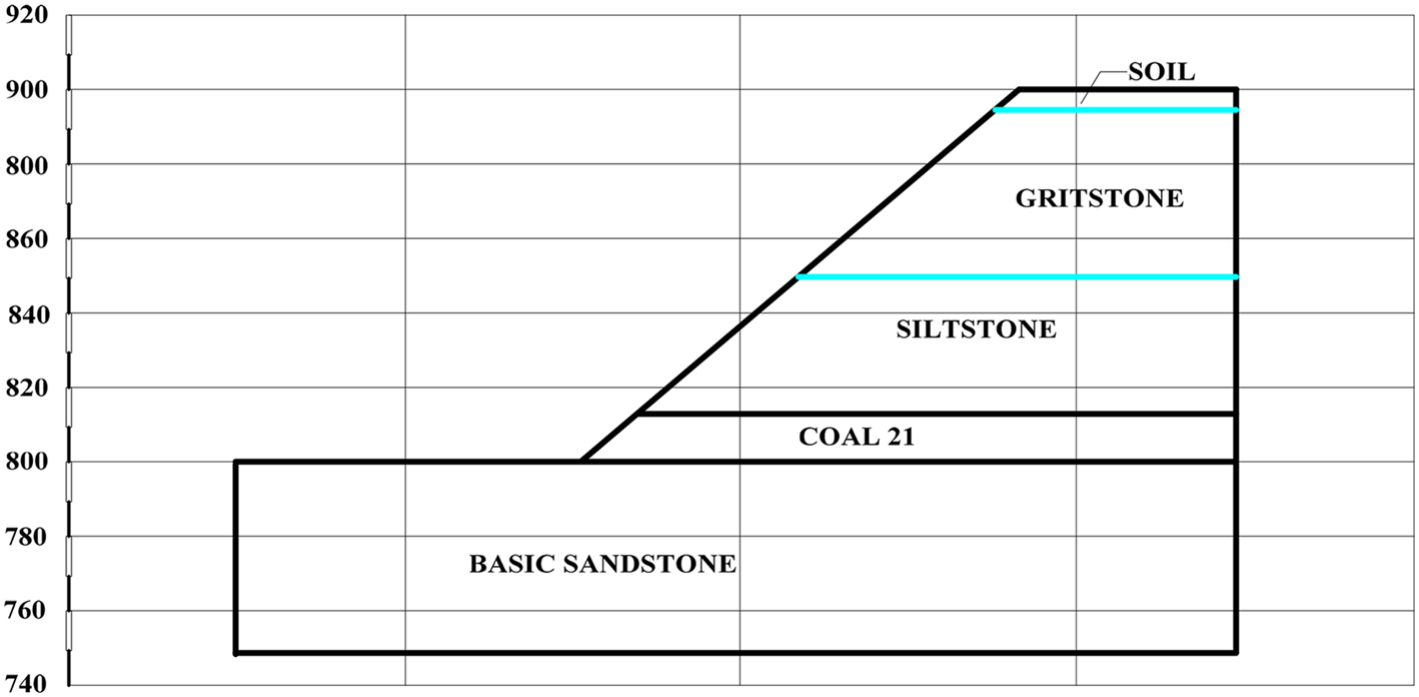
Schematic diagram of the lithology distribution for each stratum of the slope.

Samples were collected from an open-cut coal mine in Inner Mongolia with a total length of 5.1 km. The major coal seams are nos. 19 and 21 with a current mining rate of 360 m/a. The no. 21 coal seam must be rapidly extracted to enable in-pit dumping with a dumping track distance of 50 m. The dip of the no. 21 coal seam floor is relatively flat with underlying competent basal sandstone. Figure 1 shows the lithology of the pit. An LDC100 highwall mining machine (Liaoning Hanshi Mechanical Manufacture Co. Ltd.) was used to extract the coal seam from the western toe of the highwall at a production rate of 60 t/h, which is suitable for a coal stiffness value of less than 2. The undercover depth of the shovel plate is 100 mm, the undercover cutting depth is 200 mm, the traction power is 75,000 N·m, and the loading capacity is 2.6 m^3^/min. The cross-section of the highwall channel is rectangular with a width of 2 m and height of 2.5 m. The maximum mining length is 100 m, and the retreat frequency is three days. The slope height at the western end is approximately 100 m with an overall slope angle of 38°. Table 1 lists the mechanical properties of the rock mass above and below the coal seams.

**Table 1:** Mechanical properties of rock and soil mass.

Lithology	Compressive strength/MPa	Tensile strength/MPa	Cohesion/MPa	Friction angle/°	Elasticity modulus/GPa	Poisson ratio	Density/kg·m^-3^
Basal sandstone	45.19	3.40	2.18	23	15.16	0.22	2010
No.21 Coal seam	17.66	2.06	–	–	7.41	0.29	1270
Siltstone	25.76	3.02	2.35	25	14.58	0.21	2090
Coarse sandstone	51.43	4.90	2.12	22	15.62	0.21	1990
Topsoil	9.49	1.34	0.24	20	5.69	0.3	2671

## Methods

### Test scheme

Coal has soft rheological properties and its strength decreases over time. A comprehensive understanding of coal pillar strength over time is required to ensure that the slope is sufficiently supported under highwall mining operations. The coal pillar strength at the end of the strip is assumed as the “target time strength,” which should be sufficient for slope support until the end of its life. The coal pillar strength at the 20-day mark was thus used as the basis for the initial stability analysis. The coal pillar strength was studied after 7 and 14 days to consider mining rate variations at different sites.

Shear tests were first performed to determine the shear strength of the specimens. Time-dependency tests were then performed to allow the specimens to fail on the 7th, 14th, and 20th days. Each test series was repeated three times under four different normal stress conditions (0.6, 0.8, 1.0, and 1.2 MPa) to minimize bias in the results.

**Figure 2:**
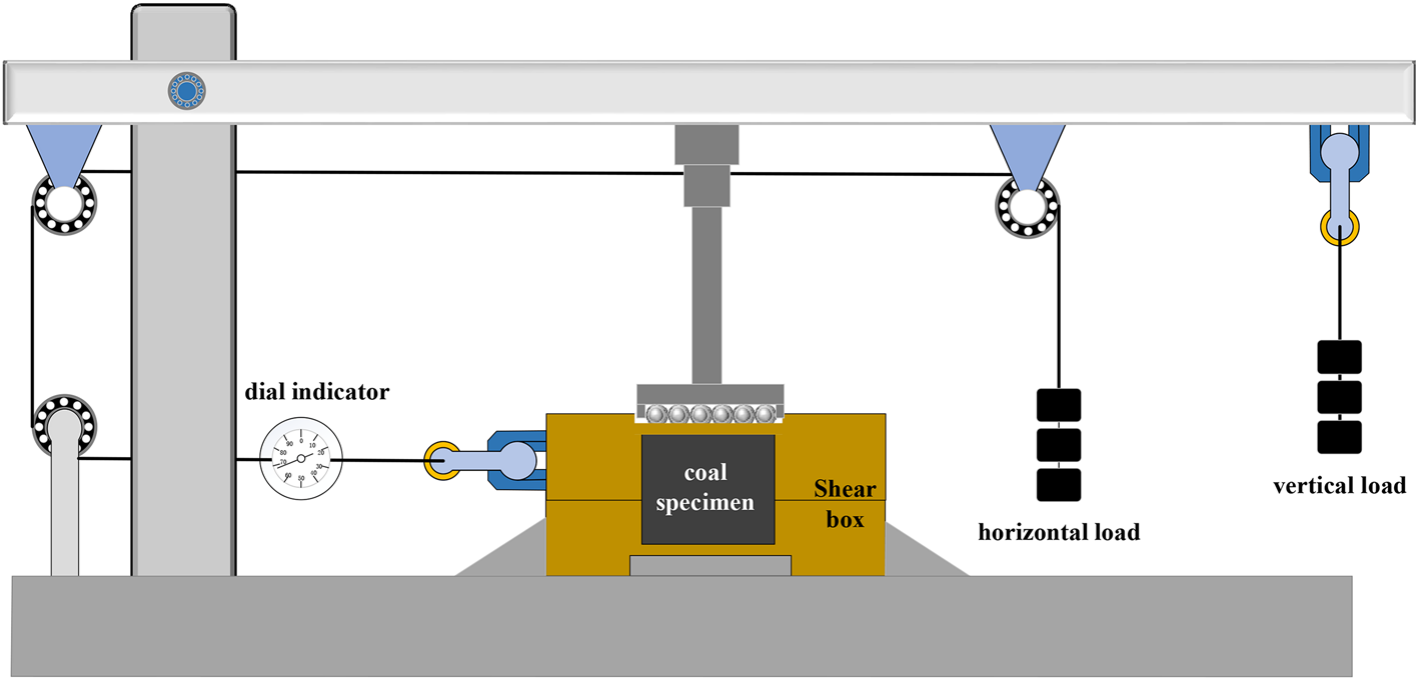
Soft rock shear creep testing machine for the lever gravity load.

A customized shear box was used for the time-dependency shear tests to facilitate the preparation of soft rock specimens with specified dimensions (Fig. 2). The specimens were initially collected in situ, prepared into volumes of 50 mm^3^, placed in a shear box, and the space between the specimen and box was then filled with cement. The normal shear testing procedure was then performed with a shear displacement measurement accuracy of 0.001 mm.

### Time-dependent behavior of coal pillar strength

1. Direct shear test results
  After placing the specimens in the box, a normal stress was applied and held constant while increasing the shear stress until failure. The shear strength was obtained as the average of three tests, which were determined under normal stress conditions of 0.766, 0.840, 0.907, and 0.980 MPa.
  According to the Mohr–Coulomb failure criterion, shear stress on a failure surface is a function of the normal stress on the surface and exhibits a curve in the associated coordinates. The Mohr envelope represents the relationship between the slip surface and normal stress when the material is subjected to different horizontal stresses and reaches the limit state. Both theoretical analysis and experiments show that the Mohr envelope can be approximately replaced by a straight line. The equation of the line is Coulomb’s formula, and the Mohr strength equation is:
  1 $$\tau = c + \sigma \tan \varphi$$
  where *τ* is the shear stress of the rock mass (MPa), *σ* is the normal stress (MPa), *c* is the cohesion (MPa), and *φ* is angle of internal friction (°).
  Fitting the direct shear test results based on the Mohr–Coulomb failure criterion yields the following fitting equation:
  2 $$\tau = 0.56 + 0.36\sigma$$
  where *c* = 0.56 MPa and *φ* = 19.8°.
2. Shear strength on the 7th day
  A stepped loading method is applied in the time-dependency shear tests. The loads at each level are set based on the shear strength of the coal specimens obtained from the direct shear tests and the variation trend of the displacement curves during loading. The coal specimen is guaranteed to not be damaged in the first 6 days, and the 7-day target time shear strength of the coal specimen is obtained once damage occurs on the 7th day. The load prior to the failure shear stress is taken as the target time strength of the specimen, and the average horizontal stress of the three specimens is taken as the shear strength under the normal stress of the associated grade.
  Figure 3 shows the deformation-time curve under various normal stress conditions for different shear stress levels on the 7th day. The shear strengths are 0.74, 0.81, 0.88, and 0.95 MPa at normal stresses of 0.6, 0.8, 1, and 1.2 MPa, respectively.

**Figure 3:**
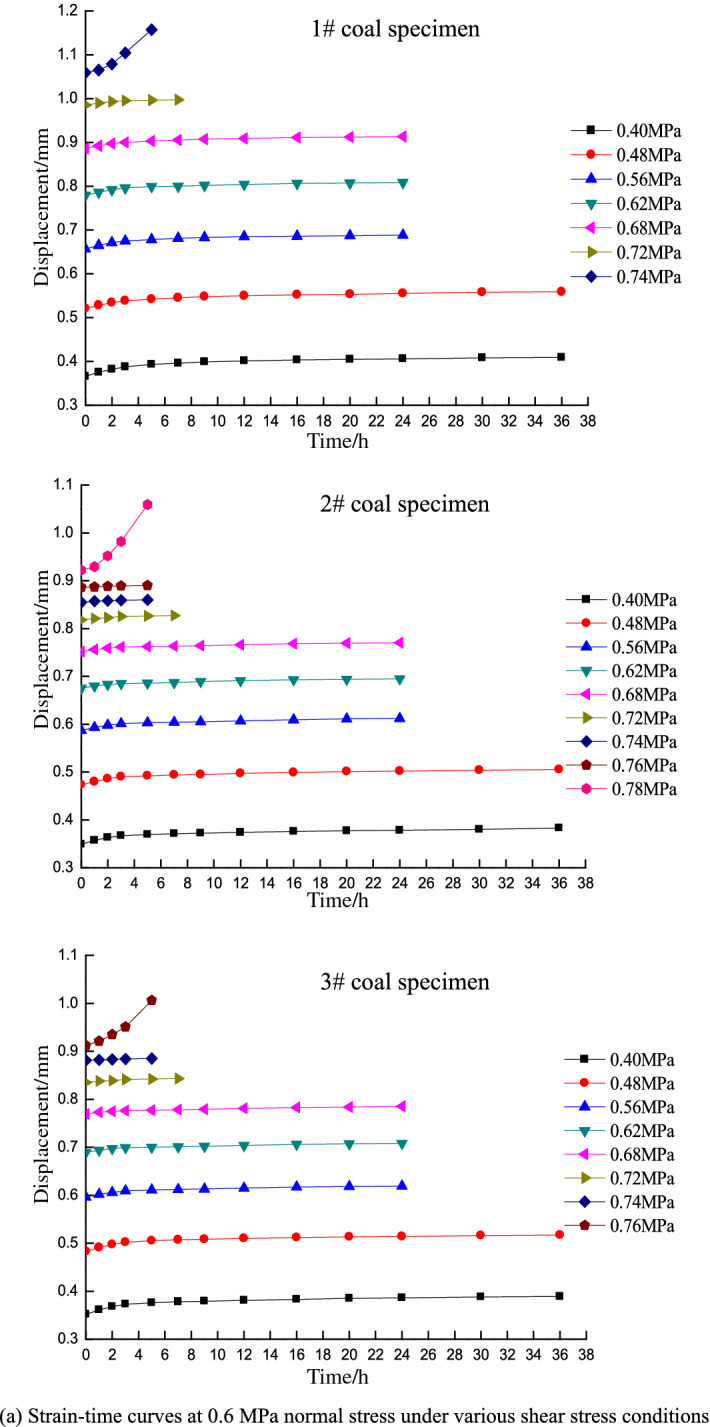

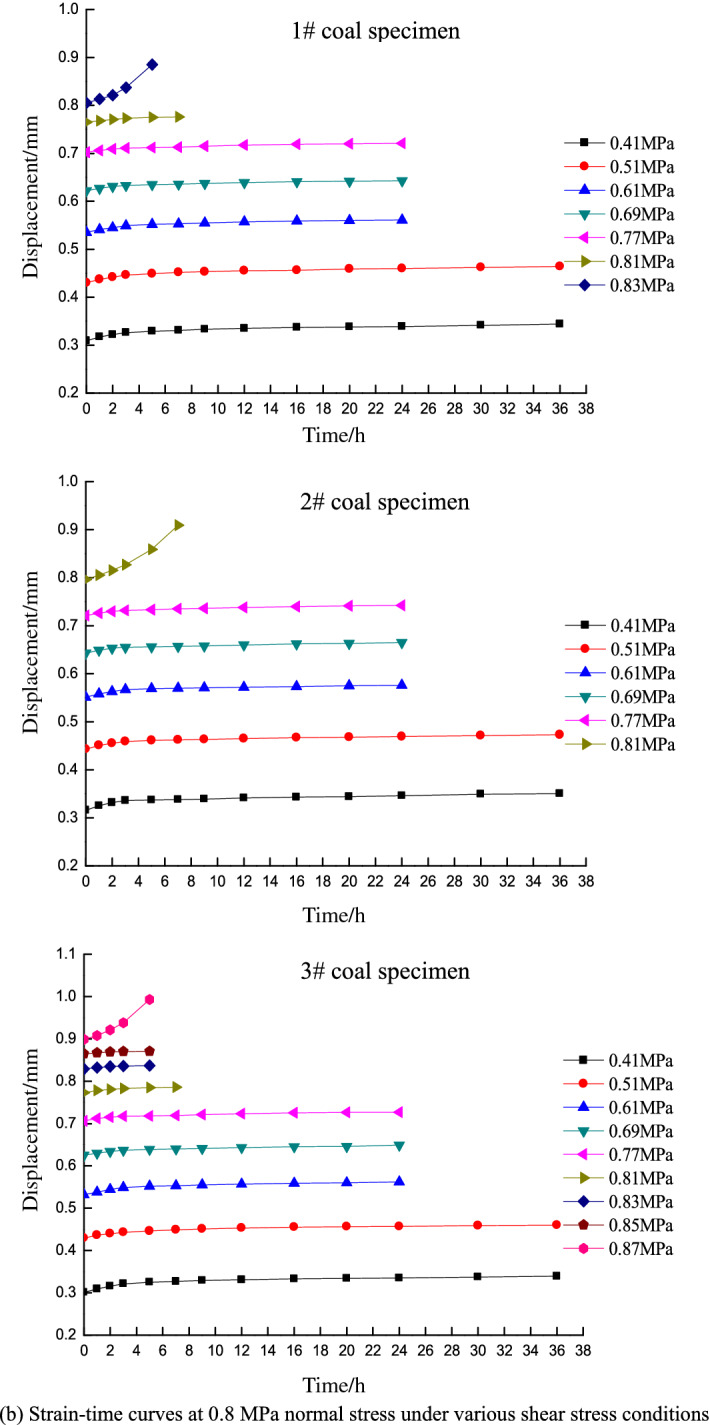

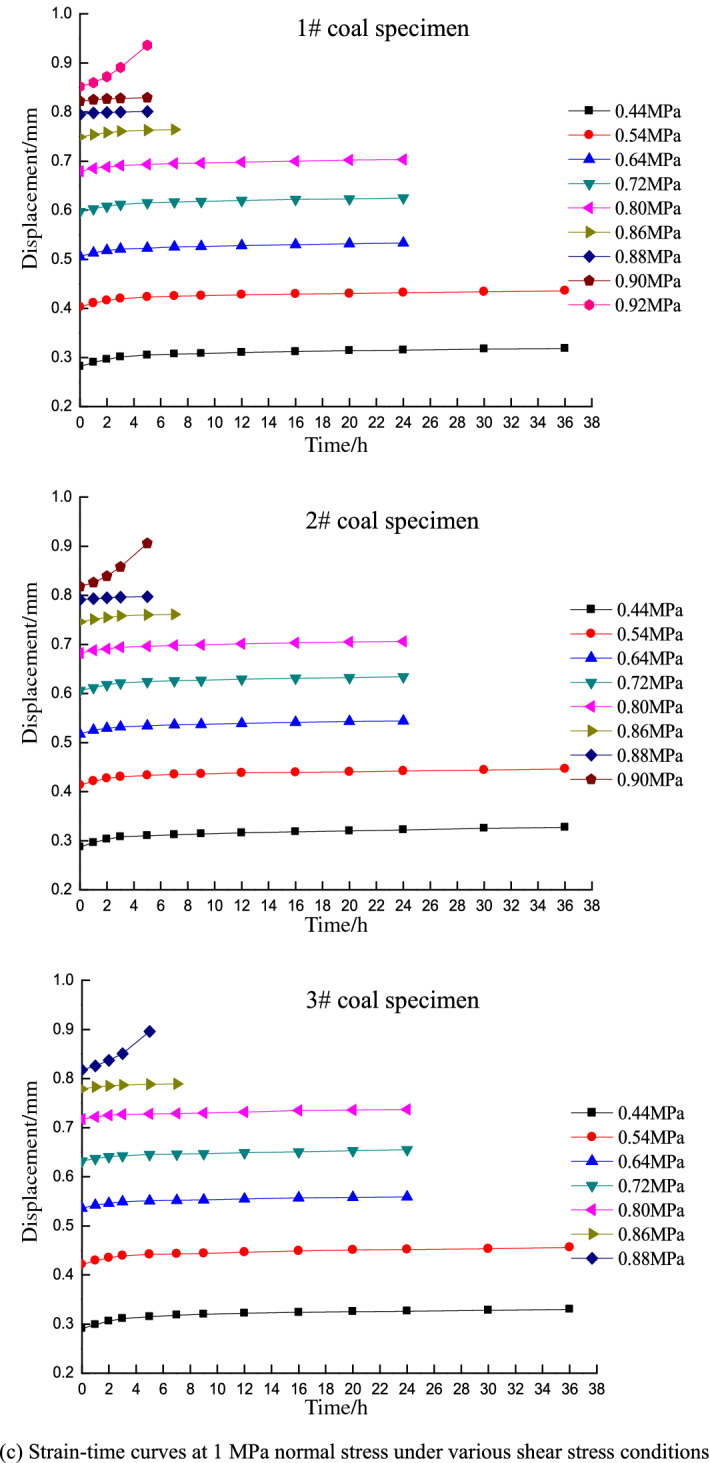

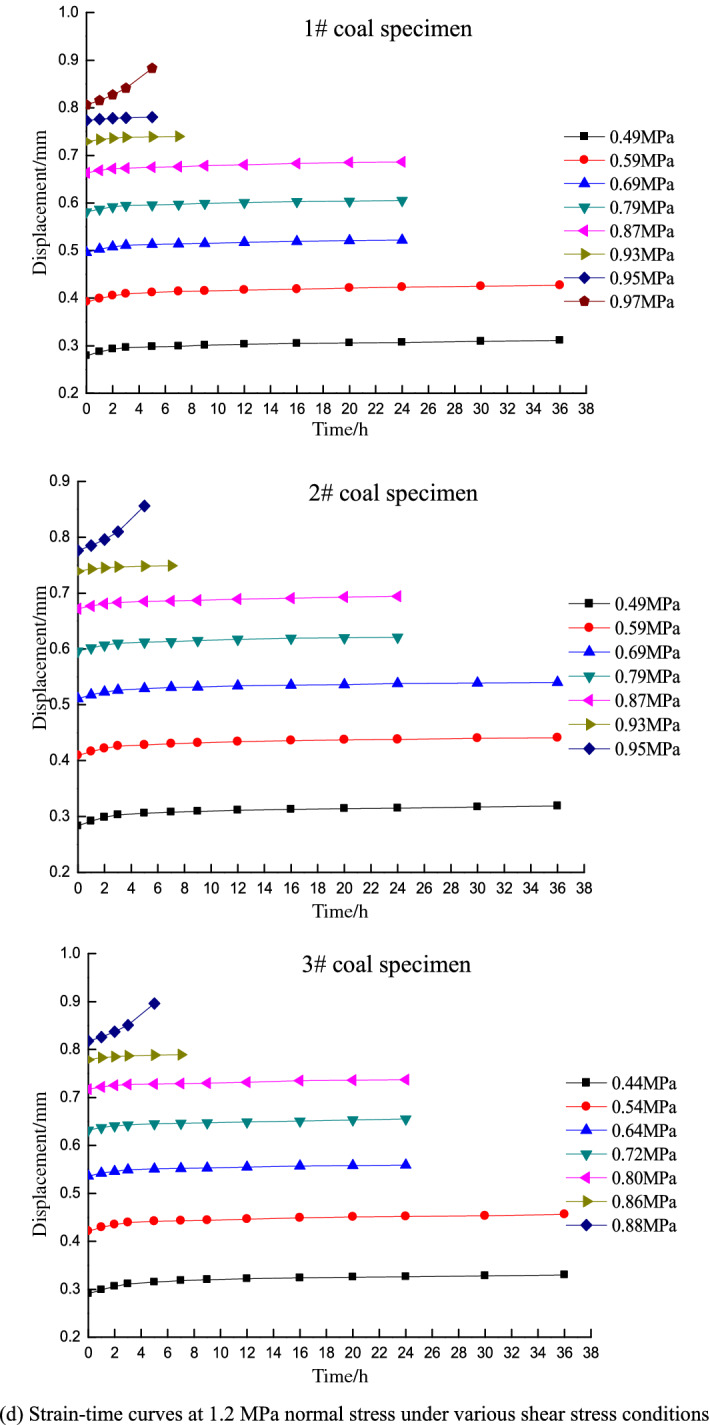
Strain–time curves of different shear stresses under various normal stress conditions for the 7-day tests.
3. Shear strength on the 14th day
  As above, the loads at each level are set based on the shear strength of coal specimens obtained from the direct shear tests and the variation trend of displacement curves during loading. In this case, the coal specimen is not damaged in the first 13 days, and the 14-day target time shear strength of the coal specimen is obtained when damage occurs on the 14th day. The shear strengths are 0.71, 0.78, 0.85, and 0.92 MPa at normal stresses of 0.6, 0.8, 1, and 1.2 MPa on the 14th day, respectively.
4. Shear strength on the 20th day
  In the 20-day tests, the loads are set following the same procedure as that in the 7- and 14-day tests, and the coal specimen is not damaged in the first 19 days. The 20-day target time shear strength of the coal specimen is obtained when damage occurs on the 20th day. The shear strengths are 0.68, 0.75, 0.81, and 0.88 MPa at normal stresses of 0.6, 0.8, 1, and 1.2 MPa on the 20th day, respectively.
  Figure 3 indicates that the strain–time curves only exhibit primary (transient deformation rate) and secondary (steady deformation rate) creep stages under low shear stress conditions, whereas the tertiary creep stage (accelerated deformation rate) is not observed. The specimens did not fail under these circumstances and the deformation converged to a given value. In contrast, the specimens underwent primary creep and subsequently entered the tertiary stage until failure under high shear stress conditions. This process only lasts for a short time of approximately 5 h. The creep curve thus increases exponentially.

**Figure 4:**
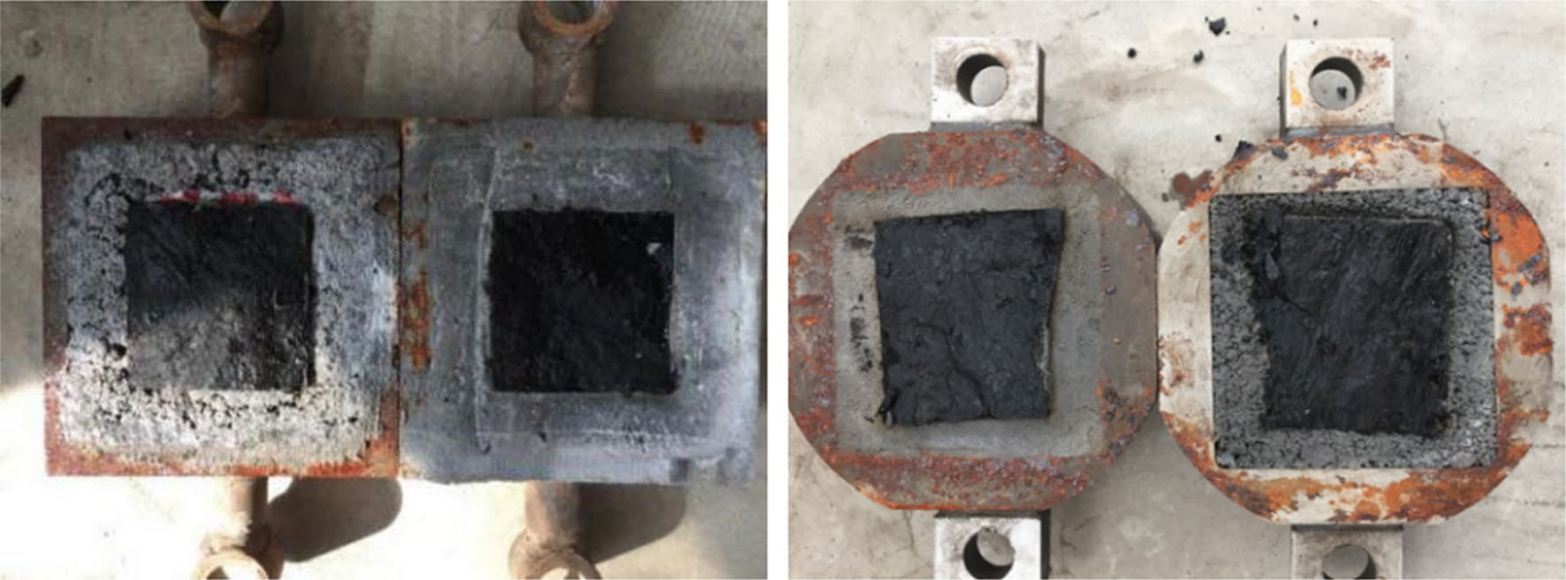
Shear creep failure planes of the coal samples.

The experimental results indicate that cracks form and expand in the coal specimens during the shear creep process. Microcracks appear upon increasing the shear stress level, which slowly expand and stabilize. If the shear stress continues to increase, the microcracks continue to expand until forming a complete shear fracture surface once the last grade of the shear stress is applied, followed by failure. Figure 4 reveals that the failure surface is relatively flat with some scratches. This implies that microcracks develop and accumulate within the specimen to induce friction between the two sides during creep. The creep behavior of the coal specimen also exhibits discreteness, which is related to the shear failure plane, particle distribution, and microcracks.

The Mohr–Coulomb parameters for the 7th, 14th, and 20th days are estimated as:

3 $$\tau = 0.53 + 0.35\sigma$$

4 $$\tau = 0.51 + 0.34\sigma$$

5 $$\tau = 0.48 + 0.335\sigma$$

where *c*_7_ = 0.53 MPa and *φ*_7_ = 19.3°, *c*_14_ = 0.51 MPa and *φ*_14_ = 18.8°, and *c*_20_ = 0.48 MPa and *φ*_20_ = 18.5°.

**Figure 5:**
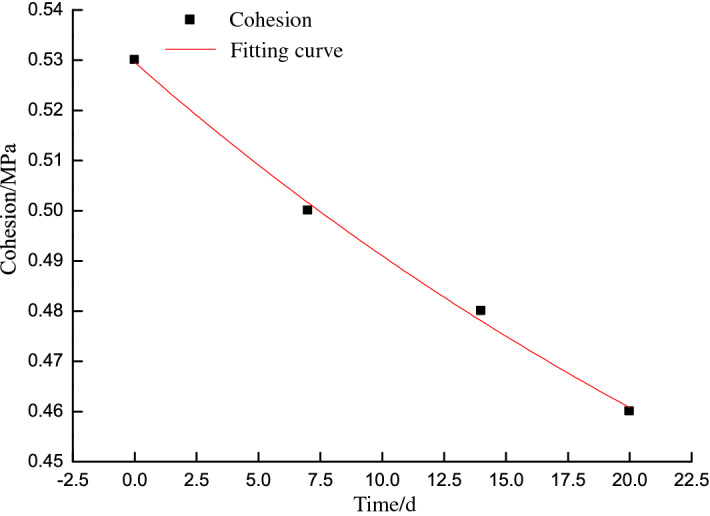
Cohesion versus time.

**Figure 6:**
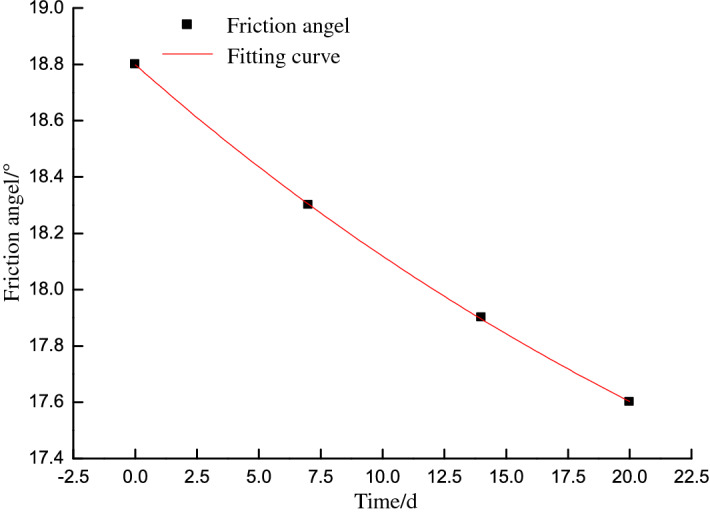
Friction angle versus time.

The mechanical properties of the slope mass are derived from the specimen properties with a reduction factor based on the Technical Code for Building Slope Engineering^22^. An investigation of the western highwall toe indicated no notable fracture development at the coal seam for an assumed reduction factor of 0.95. The cohesion and friction angle were thus respectively reduced to 0.53 MPa and 18.8° for the normal shear test, 0.5 MPa and 18.3° for the 7-day shear test, 0.48 MPa and 17.9° for the 14-day shear test, and 0.46 MPa and 17.6° for the 20-day shear test. The coal pillar shear strength parameters decrease exponentially for shallow coal seams, as shown in Figs. 5 and 6.

The time-dependent cohesion and friction angle can be expressed as:

6 $$c_{t} = 0.18e^{-0.00243t} + 0.35$$

where *c*_*t*_ is the time-dependent cohesion (MPa) and *t* is the target time (h).

7 $$\varphi_{t} = 2.84e^{-0.02735t} + 15.96$$

where *φ*_*t*_is the time-dependent friction angle (°).

## Investigation of slope stability calculation method in highwall mining

**Figure 7:**
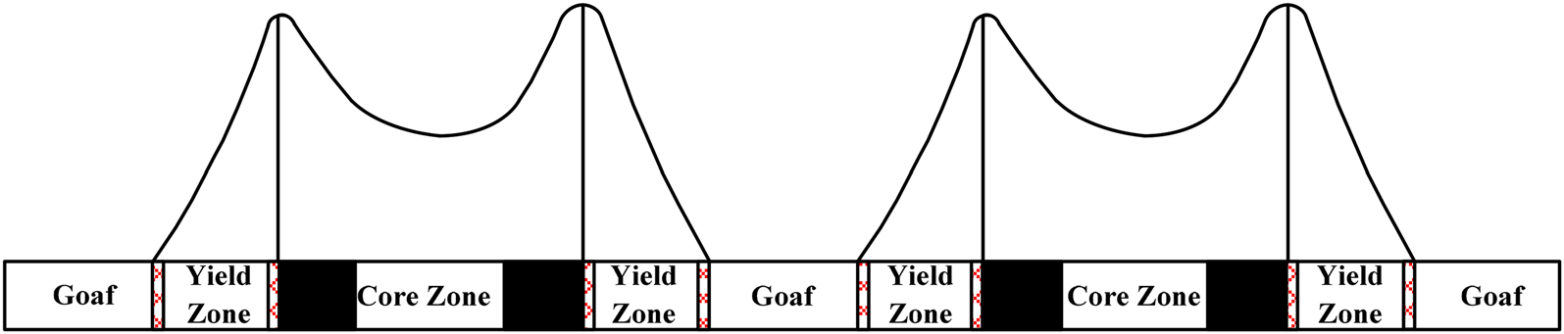
High mining area division.

Coal pillars are left between highwall channels to ensure stability during highwall mining. Based on discontinuous structural plane theory^21^, the highwall mining area can be considered as a discontinuous structural plane that consists of a goaf and coal pillar. This coal seam can become a weak layer under the weight of overlying strata, which changes the potential slope failure mode from circular to sliding along the weak plane. The weak coal seam layer can be divided into the goaf, yielding, and elastic zones, as shown in Fig. 7. The yielding and elastic zones both provide shear resistance during slope sliding. The proportions of the contact surface of the goaf, yielding, and elastic zones along the sliding surface are defined as *K*_1_, *K*_2_, and *K*_3_, respectively. The overall shear resistance can be expressed as:

8 $$\tau = K_{1} C_{r} + K_{2} C_{p} + K_{3} C_{e} + \sigma (K_{1} \tan \varphi_{r} + K_{2} \tan \varphi_{p} + K_{3} \tan \varphi_{e} )$$

where *C*_*r*_, *C*_*p*_, and *C*_*e*_ represent the cohesion values in the goaf, yielding, and elastic zones (MPa), respectively, and *φ*_*r*_, *φ*_*p*_, and *φ*_*e*_ represent the friction angles of the goaf, yielding, and elastic zones (°). The *C*_*r*_, *φ*_*r*_, and *C*_*p*_ values are negligible and assumed to be zero, whereas *φ*_*p*_ = *φ*_*e*_, *C*_*e*_ = *C*, and *φ*_*e*_ = *φ*.

Comparing Eq. (8) with the Mohr–Coulomb failure criterion, the *C*_*w*_ and *φ*_*w*_ values of the weak coal strata can be calculated as:

9 $$C_{w} = K_{1} C_{r} + K_{2} C_{p} + K_{3} C_{e}$$

10 $$\tan \varphi_{w} = K_{1} \tan \varphi_{r} + K_{2} \tan \varphi_{p} + K_{3} \tan \varphi_{e}$$

**Figure 8:**
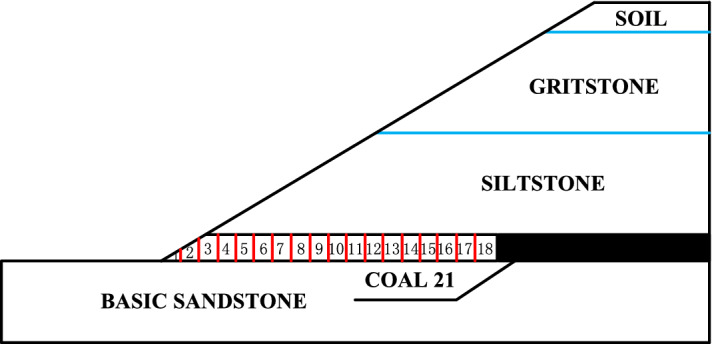
Schematic diagram of the coal pillar numbers.

Based on the “triangular load” of the slope, the coal pillar capacity should increase with mining length. The target time strength should therefore gradually increase owing to the shorter coal pillar exposure. Based on the interactions between the coal pillar strength and target time strength, the yielding zone widths on the coal pillar rib should vary with mining length. The capability of the highwall mining machine indicates that 3 days are required to complete a 100 m long highwall channel. A numerical model was constructed in FLAC^3D^ to investigate the plastic zone development at the coal pillar rib. The model was divided into 18 slices based on the 4 h advancement rate (5.5 m), as shown in Fig. 8. Based on the design parameter determination from ref.^9^, the minimum pillar width should be greater than or equal to 5.4 m for a 38° overall slope angle.

**Figure 9:**
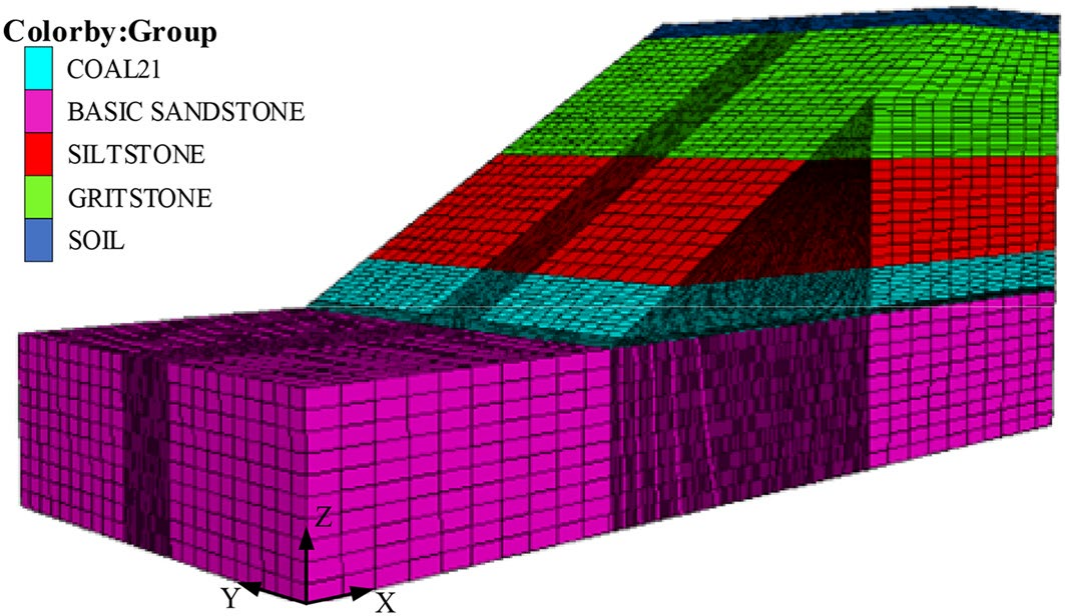
Numerical model of the slope.

Seven model cases were constructed with pillar widths between 5.4 and 6.6 m to study the changes of pillar capacity and yielding width under various loading conditions and times. The model was established based on lithology. A 60 m wide barrier pillar was left between the highwall channels to mitigate the boundary effects. The mesh size has a considerable influence on the computed results. The mesh density was thus set to increase near the coal pillar to 1 m along the pillar direction, whereas 10 × 10 nodes were covered for the cross-section of the barrier pillar. The model boundary conditions were assumed to be zero horizontal movement on the left and right boundaries with a fixed bottom boundary, whereas the top and slope face boundaries were assumed to be free-face. The Mohr–Coulomb model was used for simulations with gravity loading. Figure 9 shows a schematic view of the model.

Substituting *t* at each slice into Eqs. (6) and (7) provides *C*_*t*_and *φ*_*t*_. The peak load (*P*_*d*_) location and support capacity (*σ*_*z*_) at each coal slice can be obtained from the numerical results. The *σ*_*z*_ value can then be used to calculate the yielding zone width (*x*_*p*_)^23,24^ and subsequently calculate the yielding width of each coal slice with different pillar widths. The results allow the goaf, yielding, and elastic coefficients (*K*_1_, *K*_2_, and *K*_3_) to be estimated for the 500 m long extraction. Combining the target time (*t*), mining length (*L*), and *P*_*d*_ allows the *C*_*t*_, *φ*_*t*_, *x*_*p*_, *C*_*w*_, and *φ*_*w*_ values to be estimated, as shown in Tables 2, 3, 4, 5, 6, 7 and 8. The influence of loading was generally greater on the yielding zone width than the target time strength. The loading increased with increasing mining length, which contributes to a larger yielding zone width. The yielding zone width also decreases with increasing pillar width.

11 $$x_{p} = \ln \left[\frac{{\frac{{2(\gamma H\tan \varphi_{0} + C_{0} )}}{{(NA\tan \varphi_{0} + C_{0} )}}X + \frac{{w_{m} \gamma H\tan^{2} \varphi_{0} }}{{h(NA\tan \varphi_{0} + C_{0} )}} + 1}}{(X + 1)}\right]$$

where $$X = { 2}A{\text{tan}}\varphi_{0} x_{p} h$$, $$A = \left( {{1 } + {\text{ sin}}\varphi } \right)/\left( {{1 } - {\text{ sin}}\varphi } \right)$$, $$N = \left( {{2}c{\text{cos}}\varphi } \right)/\left( {{1 } + {\text{ sin}}\varphi } \right),\;C_{0} = C_{t} ,{\text{ and}}\varphi_{0} = \varphi_{{\text{t}}} .$$

**Table 2:** Slice width distribution pattern and evolution weak layer parameters of a 5.4-m-wide coal pillar.

Time/h	4	8	12	16	20	24	28	32	36
Chamber depth/m	5.5	11	16.5	22	27.5	33	38.5	44	49.5
*P*_*d*_	2.2	7.5	12.8	18	23.5	28.9	34.2	39.5	44.9
*C*_*t*_*/*MPa	0.461	0.462	0.462	0.463	0.463	0.464	0.464	0.465	0.465
*φ*_*t*_/°	17.61	17.62	17.63	17.63	17.64	17.65	17.66	17.66	17.67
*σ*_*z*_*/*MPa	0.096	0.116	0.146	0.188	0.241	0.307	0.376	0.448	0.547
*x*_*p*_/m	0.1	0.12	0.15	0.19	0.24	0.3	0.36	0.42	0.5
*C*_*w*_*/*MPa	0.323	0.321	0.317	0.313	0.307	0.3	0.292	0.285	0.275
*φ*_*w*_/°	12.8	12.81	12.82	12.82	12.83	12.83	12.84	12.84	12.85
Time/h	40	44	48	52	56	60	64	68	72
Chamber depth/m	55	60.5	66	71.5	77	82.5	88	93.5	100
*P*_*d*_	50	55.5	60.9	66.2	71.5	76.9	82.2	87.5	93.8
*C*_*t*_*/*MPa	0.466	0.466	0.467	0.467	0.468	0.468	0.469	0.469	0.470
*φ*_*t*_/°	17.68	17.69	17.70	17.70	17.71	17.72	17.73	17.74	17.75
*σ*_*z*_*/*MPa	0.653	0.777	0.940	1.131	1.355	1.619	1.928	2.293	2.887
*x*_*p*_/m	0.58	0.67	0.78	0.9	1.03	1.17	1.32	1.48	1.71
*C*_*w*_*/*MPa	0.266	0.255	0.241	0.226	0.21	0.193	0.174	0.154	0.125
*φ*_*w*_/°	12.85	12.86	12.87	12.87	12.88	12.88	12.89	12.9	12.9

**Table 3:** Slice width distribution pattern and evolution weak layer parameters of a 5.6-m-wide coal pillar.

Time/h	4	8	12	16	20	24	28	32	36
Chamber depth/m	5.5	11	16.5	22	27.5	33	38.5	44	49.5
*P*_*d*_	2.2	7.5	12.8	18	23.5	28.9	34.2	39.5	44.9
*C*_*t*_*/*MPa	0.461	0.462	0.462	0.463	0.463	0.464	0.464	0.465	0.465
*φ*_*t*_/°	17.61	17.62	17.63	17.63	17.64	17.65	17.66	17.66	17.67
*σ*_*z*_*/*MPa	0.086	0.106	0.136	0.177	0.219	0.285	0.353	0.423	0.522
*x*_*p*_/m	0.09	0.11	0.14	0.18	0.22	0.28	0.34	0.40	0.48
*C*_*w*_*/*MPa	0.327	0.326	0.322	0.318	0.313	0.306	0.299	0.293	0.283
*φ*_*w*_/°	12.93	12.93	12.94	12.94	12.95	12.96	12.96	12.96	12.97
Time/h	40	44	48	52	56	60	64	68	72
Chamber depth/m	55	60.5	66	71.5	77	82.5	88	93.5	100
*P*_*d*_	50	55.5	60.9	66.2	71.5	76.9	82.2	87.5	93.8
*C*_*t*_*/*MPa	0.466	0.466	0.467	0.467	0.468	0.468	0.469	0.469	0.470
*φ*_*t*_/°	17.68	17.69	17.70	17.70	17.71	17.72	17.73	17.74	17.75
*σ*_*z*_*/*MPa	0.626	0.749	0.894	1.066	1.285	1.522	1.822	2.175	2.751
*x*_*p*_/m	0.56	0.65	0.75	0.86	0.99	1.12	1.27	1.43	1.66
*C*_*w*_*/*MPa	0.274	0.263	0.251	0.237	0.222	0.206	0.188	0.168	0.14
*φ*_*w*_/°	12.98	12.98	12.99	12.99	13	13.01	13.01	13.02	13.03

**Table 4:** Slice width distribution pattern and evolution weak layer parameters of a 5.8-m-wide coal pillar.

Time/h	4	8	12	16	20	24	28	32	36
Chamber depth/m	5.5	11	16.5	22	27.5	33	38.5	44	49.5
*P*_*d*_	2.2	7.5	12.8	18	23.5	28.9	34.2	39.5	44.9
*C*_*t*_*/*MPa	0.461	0.462	0.462	0.463	0.463	0.464	0.464	0.465	0.465
*φ*_*t*_/°	17.61	17.62	17.63	17.63	17.64	17.65	17.66	17.66	17.67
*σ*_*z*_*/*MPa	0.076	0.096	0.126	0.157	0.198	0.252	0.319	0.388	0.472
*x*_*p*_/m	0.08	0.10	0.13	0.16	0.20	0.25	0.31	0.37	0.44
*C*_*w*_*/*MPa	0.332	0.330	0.327	0.324	0.319	0.314	0.307	0.3	0.292
*φ*_*w*_/°	13.05	13.06	13.06	13.06	13.07	13.08	13.09	13.09	13.09
Time/h	40	44	48	52	56	60	64	68	72
Chamber depth/m	55	60.5	66	71.5	77	82.5	88	93.5	100
*P*_*d*_	50	55.5	60.9	66.2	71.5	76.9	82.2	87.5	93.8
*C*_*t*_*/*MPa	0.466	0.466	0.467	0.467	0.468	0.468	0.469	0.469	0.470
*φ*_*t*_/°	17.68	17.69	17.70	17.70	17.71	17.72	17.73	17.74	17.75
*σ*_*z*_*/*MPa	0.573	0.693	0.835	1.002	1.232	1.446	1.739	2.083	2.644
*x*_*p*_/m	0.52	0.61	0.71	0.82	0.96	1.08	1.23	1.39	1.62
*C*_*w*_*/*MPa	0.283	0.273	0.261	0.248	0.232	0.218	0.2	0.181	0.153
*φ*_*w*_/°	13.1	13.11	13.12	13.12	13.12	13.13	13.14	13.15	13.15

**Table 5:** Slice width distribution pattern and evolution weak layer parameters of a 6.0-m-wide coal pillar.

Time/h	4	8	12	16	20	24	28	32	36
Chamber depth/m	5.5	11	16.5	22	27.5	33	38.5	44	49.5
*P*_*d*_	2.2	7.5	12.8	18	23.5	28.9	34.2	39.5	44.9
*C*_*t*_*/*MPa	0.461	0.462	0.462	0.463	0.463	0.464	0.464	0.465	0.465
*φ*_*t*_/°	17.61	17.62	17.63	17.63	17.64	17.65	17.66	17.66	17.67
*σ*_*z*_*/*MPa	0.067	0.086	0.116	0.146	0.188	0.241	0.296	0.364	0.448
*x*_*p*_/m	0.07	0.09	0.12	0.15	0.19	0.24	0.29	0.35	0.42
*C*_*w*_*/*MPa	0.336	0.335	0.331	0.329	0.324	0.319	0.313	0.307	0.299
*φ*_*w*_/°	13.15	13.16	13.17	13.17	13.18	13.18	13.19	13.19	13.2
Time/h	40	44	48	52	56	60	64	68	72
Chamber depth/m	55	60.5	66	71.5	77	82.5	88	93.5	100
*P*_*d*_	50	55.5	60.9	66.2	71.5	76.9	82.2	87.5	93.8
*C*_*t*_*/*MPa	0.466	0.466	0.467	0.467	0.468	0.468	0.469	0.469	0.470
*φ*_*t*_/°	17.68	17.69	17.70	17.70	17.71	17.72	17.73	17.74	17.75
*σ*_*z*_*/*MPa	0.535	0.653	0.777	0.940	1.148	1.373	1.658	1.994	2.541
*x*_*p*_/m	0.49	0.58	0.67	0.78	0.91	1.04	1.19	1.35	1.58
*C*_*w*_*/*MPa	0.291	0.281	0.271	0.259	0.244	0.228	0.211	0.193	0.166
*φ*_*w*_/°	13.21	13.21	13.22	13.22	13.23	13.24	13.24	13.25	13.26

**Table 6:** Slice width distribution pattern and evolution weak layer parameters of a 6.2-m-wide coal pillar.

Time/h	4	8	12	16	20	24	28	32	36
Chamber depth/m	5.5	11	16.5	22	27.5	33	38.5	44	49.5
*P*_*d*_	2.2	7.5	12.8	18	23.5	28.9	34.2	39.5	44.9
*C*_*t*_*/*MPa	0.461	0.462	0.462	0.463	0.463	0.464	0.464	0.465	0.465
*φ*_*t*_/°	17.61	17.62	17.63	17.63	17.64	17.65	17.66	17.66	17.67
*σ*_*z*_*/*MPa	0.057	0.076	0.106	0.136	0.177	0.23	0.285	0.353	0.423
*x*_*p*_/m	0.06	0.08	0.11	0.14	0.18	0.23	0.28	0.34	0.4
*C*_*w*_*/*MPa	0.34	0.339	0.336	0.333	0.328	0.323	0.318	0.312	0.305
*φ*_*w*_/°	13.26	13.27	13.28	13.28	13.28	13.29	13.3	13.3	13.31
Time/h	40	44	48	52	56	60	64	68	72
Chamber depth/m	55	60.5	66	71.5	77	82.5	88	93.5	100
*P*_*d*_	50	55.5	60.9	66.2	71.5	76.9	82.2	87.5	93.8
*C*_*t*_*/*MPa	0.466	0.466	0.467	0.467	0.468	0.468	0.469	0.469	0.470
*φ*_*t*_/°	17.68	17.69	17.70	17.70	17.71	17.72	17.73	17.74	17.75
*σ*_*z*_*/*MPa	0.509	0.626	0.749	0.91	1.098	1.32	1.579	1.907	2.415
*x*_*p*_/m	0.47	0.56	0.65	0.76	0.88	1.01	1.15	1.31	1.53
*C*_*w*_*/*MPa	0.298	0.288	0.278	0.265	0.252	0.238	0.222	0.204	0.179
*φ*_*w*_/°	13.31	13.32	13.33	13.33	13.34	13.34	13.35	13.36	13.37

**Table 7:** Slice width distribution pattern and evolution weak layer parameters of a 6.4-m-wide coal pillar.

Time/h	4	8	12	16	20	24	28	32	36
Chamber depth/m	5.5	11	16.5	22	27.5	33	38.5	44	49.5
*P*_*d*_	2.2	7.5	12.8	18	23.5	28.9	34.2	39.5	44.9
*C*_*t*_*/*MPa	0.461	0.462	0.462	0.463	0.463	0.464	0.464	0.465	0.465
*φ*_*t*_/°	17.61	17.62	17.63	17.63	17.64	17.65	17.66	17.66	17.67
*σ*_*z*_*/*MPa	0.047	0.067	0.096	0.126	0.167	0.209	0.263	0.33	0.40
*x*_*p*_/m	0.05	0.07	0.10	0.13	0.17	0.21	0.26	0.32	0.38
*C*_*w*_*/*MPa	0.344	0.343	0.34	0.337	0.333	0.329	0.323	0.318	0.311
*φ*_*w*_/°	13.37	13.37	13.38	13.38	13.39	13.4	13.4	13.4	13.41
Time/h	40	44	48	52	56	60	64	68	72
Chamber depth/m	55	60.5	66	71.5	77	82.5	88	93.5	100
*P*_*d*_	50	55.5	60.9	66.2	71.5	76.9	82.2	87.5	93.8
*C*_*t*_*/*MPa	0.466	0.466	0.467	0.467	0.468	0.468	0.469	0.469	0.470
*φ*_*t*_/°	17.68	17.69	17.70	17.70	17.71	17.72	17.73	17.74	17.75
*σ*_*z*_*/*MPa	0.485	0.599	0.721	0.88	1.066	1.284	1.541	1.864	2.317
*x*_*p*_/m	0.45	0.54	0.63	0.74	0.86	0.99	1.13	1.29	1.49
*C*_*w*_*/*MPa	0.307	0.294	0.285	0.272	0.26	0.245	0.23	0.212	0.191
*φ*_*w*_/°	13.42	13.43	13.43	13.43	13.44	13.45	13.46	13.46	13.47

**Table 8:** Slice width distribution pattern and evolution weak layer parameters of a 6.6-m-wide coal pillar.

Time/h	4	8	12	16	20	24	28	32	36
Chamber depth/m	5.5	11	16.5	22	27.5	33	38.5	44	49.5
*P*_*d*_	2.2	7.5	12.8	18	23.5	28.9	34.2	39.5	44.9
*C*_*t*_*/*MPa	0.461	0.462	0.462	0.463	0.463	0.464	0.464	0.465	0.465
*φ*_*t*_/°	17.61	17.62	17.63	17.63	17.64	17.65	17.66	17.66	17.67
*σ*_*z*_*/*MPa	0.038	0.057	0.086	0.116	0.157	0.198	0.252	0.319	0.376
*x*_*p*_/m	0.04	0.06	0.09	0.12	0.16	0.20	0.25	0.31	0.36
*C*_*w*_*/*MPa	0.348	0.347	0.343	0.341	0.337	0.333	0.328	0.322	0.317
*φ*_*w*_/°	13.45	13.46	13.47	13.47	13.48	13.48	13.49	13.49	13.50
Time/h	40	44	48	52	56	60	64	68	72
Chamber depth/m	55	60.5	66	71.5	77	82.5	88	93.5	100
*P*_*d*_	50	55.5	60.9	66.2	71.5	76.9	82.2	87.5	93.8
*C*_*t*_*/*MPa	0.466	0.466	0.467	0.467	0.468	0.468	0.469	0.469	0.470
*φ*_*t*_/°	17.68	17.69	17.70	17.70	17.71	17.72	17.73	17.74	17.75
*σ*_*z*_*/*MPa	0.46	0.573	0.693	0.85	1.018	1.232	1.484	1.801	2.245
*x*_*p*_/m	0.43	0.52	0.61	0.72	0.83	0.96	1.10	1.26	1.46
*C*_*w*_*/*MPa	0.31	0.3	0.291	0.279	0.268	0.253	0.239	0.222	0.2
*φ*_*w*_/°	13.51	13.52	13.52	13.52	13.53	13.54	13.55	13.55	13.56

## Parametric study of slope stability under highwall mining

**Figure 10:**
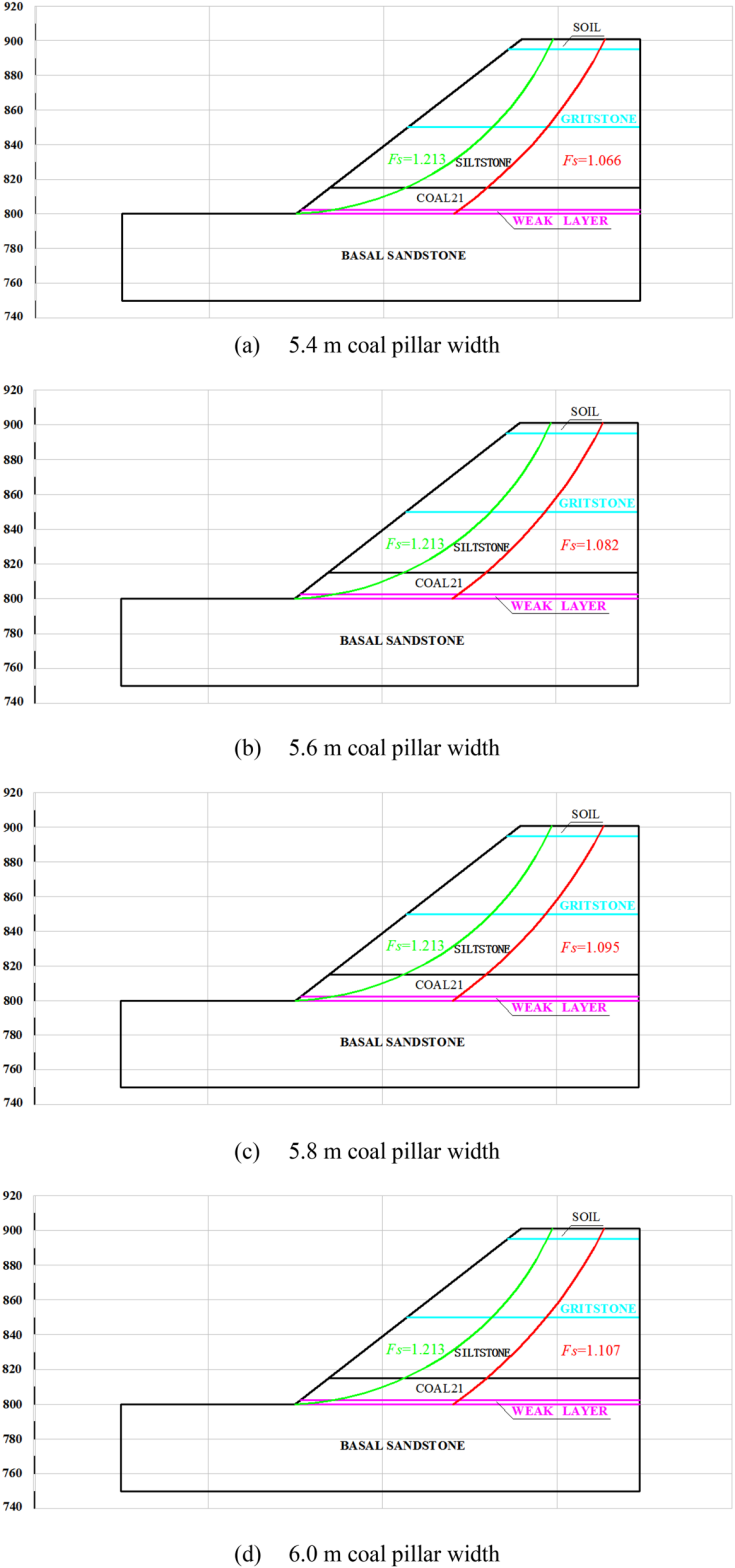

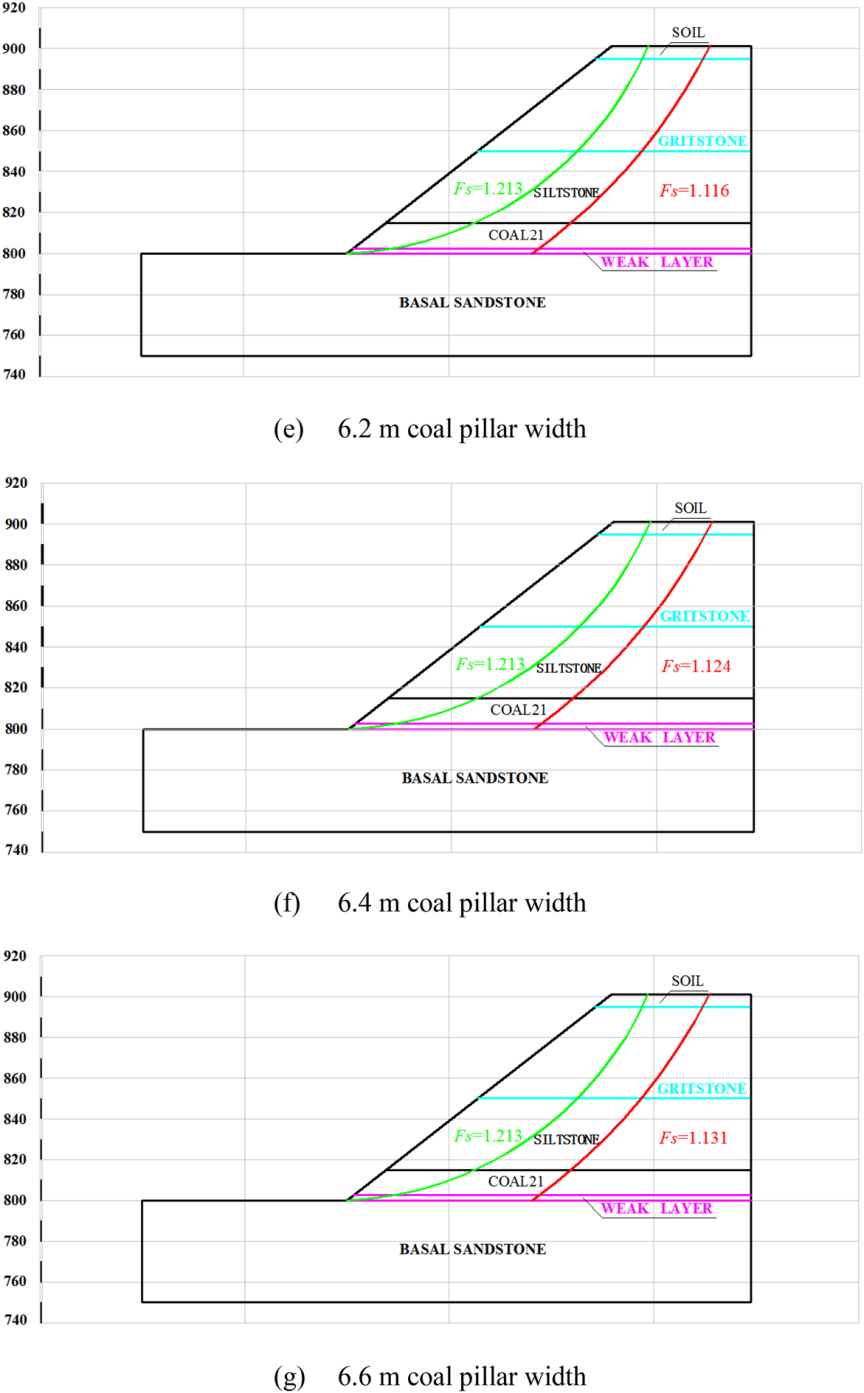
Failure paths for different coal pillar widths.

**Figure 11:**
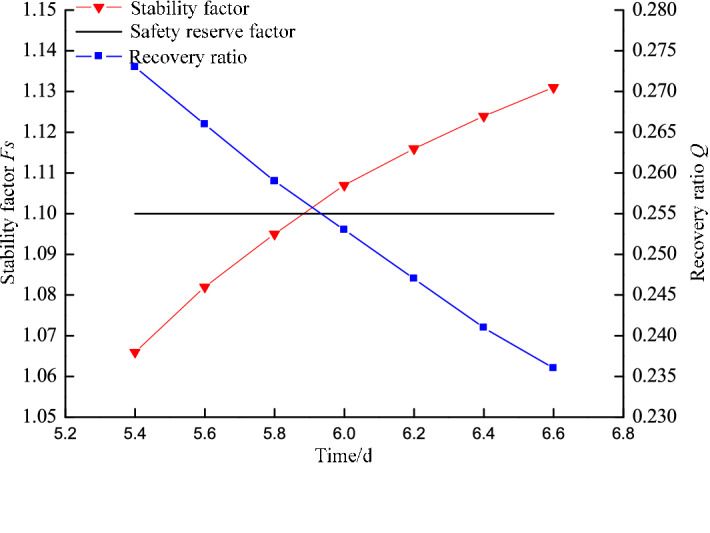
Stability coefficient and coal recovery rates for different coal pillar widths.

Based on the highwall mining conditions and current in-pit dump rate, approximately 20 days are required to complete a dump. This means the time required for the no. 21 coal seam to become a weak layer is limited. The slope stability index (*F*_*S*_) of the western highwall toe is 1.1 according to the “Code for geotechnical engineering investigation” (GB50021-2012)^25^. Independently developed software based on the rigid body-limit equilibrium method (LEM) was used to study the influence of coal pillar width on slope stability, as shown in Fig. 10. The failure mode of the slope changes from circular to slip along the coal seam. The shear-driven back-break is extended from the topsoil to the no. 21 coal seam with surface sliding along the seam. The *F*_*S*_ of the slope was found to increase with increasing collar pillar width. The *F*_*S*_ values are 1.066, 1.082, 1.095, 1.107, 1.116, 1.124, and 1.131 for pillar widths of 5.4, 5.6, 5.8, 6.0, 6.2, 6.4, and 6.6 m, respectively. The slope stability coefficient gradually increases with increasing coal pillar width, whereas the resource recovery rate gradually decreases. Figure 11 shows the recovery rate and *F*_*S*_ for different pillar widths.

Regression was used to correlate the slope stability and *F*_*S*_ to maximize the recovery rate without compromising the slope stability, according to:

12 $$F_{S} = - 10.20511e^{{( - w_{s} /1.1705)}} + 1.16721$$

where *w*_*s*_ is the coal pillar width (m), yielding a linear correlation of R^2^ = 0.99986. Only a slope stability coefficient of 1.1 can guarantee to maximize the recovered retained coal resources while maintaining safety. Substituting *F*_*S*_ = 1.1 into Eq. (12) yields 5.9 m as the optimized pillar width. However, the stability of the slope may be compromised under this pillar width threshold.

Coal in open-cut mines is generally soft with notable creep characteristics. Creep tests were performed to measure the variation of the shear strength parameters of the coal specimens with time. For a shallow buried coal seam, the shear strength parameters of the coal specimens decreased exponentially with time. However, there remains no unified consensus regarding how slope stability is affected by highwall mining largely owing to the lack of reasonable slope stability calculation methods. This study proposes a calculation method of time and space stability of a slope with mining chambers that accounts for both time and space effects. The influence of high mining on slope stability is quantified, which lays a theoretical foundation for the popularization and application of high mining technology.

## Conclusion

1. The concept of target time strength is proposed, and the time required for in-pit dumping is considered as the coal pillar service time. The pillar strength at this time is considered to be the target time strength. The time-dependent parameters of coal pillars are determined from laboratory tests. The results show that the shear strength of the coal pillar decreases exponentially with time over a certain target period.
2. The highwall mining channel is divided into goaf, yielding, and elastic zones based on discontinuous structural plane theory. This suggests that the target coal seam has the potential to become a weak layer, which can lead to changes in the slope failure mode. The numerical simulation results consider the influence of mining length and creep behavior on the plastic zone development at the pillar rib. Loading from the overlying strata is found to have a greater influence than the target time strength does on the plastic zone width. The loading increases with mining length, which results in a wider yielding zone at the coal pillar ribs. The yielding zone narrows with increasing coal pillar width. A slope stability calculation method for highwall mining is proposed using the rigid body-LEM based on the Mohr–Coulomb failure criterion.
3. The results obtained from the proposed method are used to assess the influence of highwall mining on slope stability. The slope failure mode was found to change from circular to sliding along the no. 21 coal seam. The coal pillar design was subsequently optimized to ensure slope stability while maximizing the recovery rate.
